# Sustainable strategic nation branding through sports: leveraging soft power via mega-event hosting

**DOI:** 10.3389/fsoc.2025.1521396

**Published:** 2025-03-10

**Authors:** Laila El-Dabt, Faisal AlReshaid, Kathleen Park, Nour AlBuloushi, Abrar Al-Enzi

**Affiliations:** ^1^Department of Marketing and Event Management, Australian University, West Mishref, Kuwait; ^2^Department of Management, American University of Kuwait, Safat, Kuwait; ^3^MET Department of Administrative Sciences and Global Development Policy Center, Boston University, Boston, MA, United States; ^4^Department of Management, Gulf University for Science and Technology, Mishref, Kuwait

**Keywords:** soft-power, sustainable nation branding, strategic management, sports events, Qatar, international relations

## Abstract

**Introduction:**

This study examines the strategic management of nation branding from a soft power perspective, focusing on Qatar’s use of sports mega-events to enhance its global image. In contrast to hard power, soft power, in the realm of national or public diplomacy, refers to using cooperation rather than coercion to influence the impressions and preferences of others. Our research highlights the efforts by the government of Qatar to leverage sporting events, such as the FIFA World Cup, to boost international visibility, enhance cultural exchange, and attract tourism and investments.

**Methods:**

Using document analysis and semi-structured interviews, we investigate how global sports serve as a unique soft power vehicle for nation branding, building the attraction and appeal of a nation.

**Results:**

Our findings deepen insights into how nations can strategically and sustainably use globally known and popular sports to cultivate soft power, heighten national pride, and project a positive identity on the world stage.

**Discussion:**

We contribute to the literature on soft power by developing a systematic conceptual framework for understanding how mega-sports events can be strategically managed to support nation branding and public diplomacy.

## 1 Introduction

Strategic decisions made by organizations rely on organizational capabilities to design systems that protect organizational units from external forces (Grewatsch et al., 2023). While organizations—whether firms, nations, or other collective entities—can be construed as having agency in decision-making or capability-building, such actions and capabilities ultimately derive from individuals, systems, processes or governance within the organization. Such systems enable organizations to address societal challenges effectively based on the strategic management of organizational processes (Bryson et al., 2024). Strategic management involves developing ideas, processes, and hierarchies in a manner conducive to both innovative and holistic thinking (Xia et al., 2023) and is therefore a crucial discipline at multiple levels. For example, businesses of varying sizes and as well as nations can benefit from holistic thinking guided by strong strategic intent (Bryson et al., 2024). Nations can benefit from strategic thinking rooted in a clear understanding of the processes required to support nation branding, which refers to deliberate actions to enhance the image of a nation through marketing and communication (Nicolescu and Barbu, 2024a; Nicolescu and Barbu, 2024b; Hao et al., 2021). These branding endeavors require both intent and resourcefulness, underscoring the critical role of public policymakers (Rojas-Méndez and Khoshnevis, 2023). In the branding literature, such national branding efforts resonate with normative control (Müller, 2017), which reflects the exercise of soft power (Günek, 2018).

This paper contributes to both national branding, also sometimes referred to as place branding, and strategic management literatures by exploring the mechanisms through which nations translate strategic intent into soft power initiatives. While national branding emphasizes the further improvement of the image of a country through deliberate marketing and communication efforts, strategic management focuses on the systems and processes enabling such efforts. By integrating these perspectives, we highlight how strategic management principles underpin successful nation branding practices and impact in competitive global contexts. Our dual focus provides an enhanced framework for understanding how nations use strategic capabilities to achieve soft power objectives.

Extending on the seminal work of Nye (1990), soft power is commonly understood as the ability to obtain desired outcomes through attraction and persuasion rather than coercion. Globalization has resulted in increased competition between nations and has made it imperative for governments to focus on the magnetism of a nation to attract and influence foreign audiences (Lee, 2015; Moharrak et al., 2025). Thus, the concept of soft power has gained traction in recent years as countries increasingly strive to enhance their national appeal. However, wider interest in soft power does not necessarily mean wider understanding. The definition of soft power, and its implementation, relevance, and required resources remain contested in the literature and researchers to date have not delivered a comprehensive model or explanation of the complete dimensions and implementation of soft power (Bae and Lee, 2020; Grix and Brannagan, 2016). A notable gap persists in investigation of the practical mechanisms that enable the operationalization and realization of soft power. Moreover, the relationship between soft power, nation branding, and public diplomacy remains underexplored, as existing research often treats these concepts as distinct or somewhat overlapping, but without clearly defining their interconnections. Based on these gaps in the research regarding the relationships and mechanisms underlying soft power, our study enhances understanding of how nation branding and public diplomacy contribute to the achievement of soft power. Providing a clear understanding of the concept and strategic intent behind soft power can guide managers and policymakers toward more thorough and successful implementation (Abdullahi and Othman, 2020). To clarify the interrelationship of soft power, nation branding, and public diplomacy, we examine the soft power strategy of the nation of Qatar through the hosting of sports mega-events, which provide a compelling setting for the analysis of soft power principles and practice.

## 2 Soft power, national cultures, and strategic action

### 2.1 Strategic shift from hard power to nation branding

Hard power can be a currency of world politics (Trunkos, 2013), but such coercive agents are increasingly ineffective in a rapidly evolving worldwide context dominated by social media and global culture (Kashif and Udunuwara, 2024). Modern interconnected societies enable individuals to easily share perspectives, thereby easily falsifying some traditional notions of hard power (Daßler et al., 2019). Technological advances further highlight the need for strategic changes in political approaches (Pinto, 2023). Rest apart, hard power can lead to national disasters (Desmidt and Meyfroodt, 2021; Mouazen et al., 2023), indicating a strong role of strategic management among public policymakers (Bryson et al., 2024). Moreover, this also supports the thesis of this paper that holistic and more sustainable agenda (i.e., nation branding) is possible when policymakers are understanding and implementing strategic management initiatives (Desmidt and Meyfroodt, 2021). Focused strategic action in globally popular domains helps build trust, encourage foreign investment, and strengthen global trade and bilateral agreements (Lee, 2015; Moharrak et al., 2025; Sertyesilisik, 2021), positioning nation branding and public diplomacy as a key component of sustainable national development.

We examine and seek to further provide and deepen a systematic understanding of how the hosting of mega-sport events can escalate the soft power of a nation, emphasizing strategic, political, and policy development aspects, as well as emotional and sociocultural dimensions. Previous research on mega-sports hosting from the Arabian Gulf countries or nearby emerging markets has particularly studied collective emotional impacts and long-term sociocultural transformations. For instance, Sullivan (2018) investigated the impact of the collective pride and unity generated during the South Africa 2010 FIFA World Cup to show how emotional contagion—the sharing and spread of emotions—plus the propagation of common national goals increased the sense of national agency and societal cohesion. Along similar lines, Ishac et al. (2018, 2024) and Ishac et al. (2022) revealed how the hosting of the 2022 FIFA World Cup by Qatar built community pride, attachment, and support for the Qatari 2030 national vision throughout demographic groups in the nation. Further underscoring the social and psychological dimensions, subsequent research found that these reactions of residents to mega-sport event hosting in Qatar could inform the plans for and benefits of future hosting initiatives (Al-Emadi et al., 2024). An ongoing and futuristic perspective on sport mega-event hosting underscores the sustainability perspective of such events. Also drawing on the hosting of the 2022 FIFA World Cup by Qatar, Al-Muhannadi et al. (2024) examined the evolution of innovations in environmental and cultural sustainability elicited by the international spotlight on the hosting of mega-sport events, while calling for further analysis of the long-term durability of these effects.

While the above research showed how mega-sport events advance soft power through emotional, social, and sustainability impacts, some inferences can also be drawn about policy development with respect to sustainability, community engagement, and cultural grounding. Environmental innovations such as carbon-neutral technologies and modular stadium designs that occurred as part of the Qatar 2022 FIFA hosting suggest a potential for ongoing sustainability initiatives (Al-Muhannadi et al., 2024). Results achieved in building community pride and attachment offer guidance for possible social sustainability policies to enhance resident inclusion in event planning (Ishac et al., 2018, 2022, 2024). Finally, the development and continuation of policies grounded in Gulf faith and culture while also promoting intercultural awareness and understanding can be considered as a means of maximizing both cultural sustainability and global support (Al-Emadi et al., 2024; AlShamali and AlMutairi, 2023). As there is still a need for more comprehensive frameworks for long-term soft power implementation and attendant policy development, we pursue further insights based on the hosting of mega-sport events.

### 2.2 Sports as a catalyst for nation branding

Nations can effectively leverage sports as a tool for nation branding and public diplomacy, given the integral role in sports globally in social life (AlSarraf and AlMutairi, 2025; Nicolescu and Barbu, 2024b; Kashif et al., 2019). Major sporting events attract tourists worldwide and stimulate economic growth (Vrondou, 2023), reinforcing the connection between sports and national development. Contemporary evidence suggests that hosting particularly large, international sports mega-events enhances the image of a nation and contributes positively to nation branding (Li et al., 2024). Successful event organization not only boosts national pride but also serves as a strategic example of governance branding (Gulavani et al., 2024). A case in point is the use of rugby to establish a national brand identity in various Commonwealth countries, demonstrating how government branding can revolutionize strategic thinking (Kahiya et al., 2023). This earlier outcome highlights the importance of strategic management by policymakers in using sports to advance nation branding agendas, as reflected in impressions formed by key opinion leaders as well as the general public (Vila-López and Küster-Boluda, 2024).

Nation branding extends beyond sports to include the broader concept of cultural positioning, an essential part of national brand management at the macro level (Schühly, 2022). Culture, encompassing art, music, sports, and folklore, provides what can be termed the natural identity of a nation (López Hernández, 2022). Countries such as the US, UK and Canada differentiate themselves through distinct cultural markers compared to nations in the Middle East or Africa, reflecting varying national agendas. Hofstede’s (2011) cultural dimensions model—considering of factors such as individualism, risk tolerance, and power distance—provides a framework to analyze national identity. Additionally, elements such as local heritage, language, and literature contribute to national cultural branding (Albuloushi and Algharaballi, 2014; AlMutairi and Yen, 2022; Brewer and Venaik, 2012; López Hernández, 2022). Strategic management of these cultural assets is crucial for projecting a cohesive, attractive, and appealing national image.

Mega-sport events can play a pivotal role in nation branding and public diplomacy by building the national image alongside strengthening emotional and social connections. Sullivan (2018) demonstrated how collective emotions such as pride and unity, generated during mega-events, contribute to a sense of national transformation, aligning with nation branding goals. Ishac et al. (2018, 2024) and Al-Muhannadi et al. (2024) highlighted how hosting events such as the FIFA World Cup intensifies community attachment while also exhibiting sustainability initiatives, upselling the appeal of Qatar to international audiences. It is furthermore valuable to encompass various resident and visitor groups in shaping perceptions critical for effective public diplomacy (Al-Emadi et al., 2024) and to encourage foreign investments into new products through trust (Al Reshaid et al., 2024). In these ways and more, mega-sport events have the power and potential to project soft power and advance both nation branding and public diplomacy objectives.

### 2.3 Political values and global collaboration

In addition to national culture, political and moral norms and values also play a key role. Modern political systems are evaluated by other nations through public media, tourism, and sports events (Sertyesilisik, 2021). Foreign policy also remains a cornerstone of modern nation branding, reflecting state collaboration strategies and international goals (Northedge, 1968). Globalization has further amplified the importance of cooperation, with countries increasingly coordinating interests and pooling resources to achieve shared, desired outcomes (McClory and Harvey, 2016). These efforts highlight the need for public policymakers to integrate political, economic, and cultural strategies into holistic nation branding, public diplomacy and soft power initiatives.

Holistic thinking among policymakers is critical for the successful execution of nation branding agendas, particularly through sports event organization. For instance, the Olympics in China exemplify strategic governance, showcasing the potential of well-orchestrated global events to reshape international perceptions (Diodato and Strina, 2023). Similarly, efforts by South Korea to host sports events have created strong emotional bonds with international audiences, elevating the national brand (Kim and Kim, 2024). In Africa, strategic sports event organization has countered negative stereotypes and helped to create a more positive image of the continent (Quansah, 2024). These examples demonstrate that sports-based nation branding requires substantial effort as well as a well-rounded strategic approach. Ultimately, such initiatives underscore the importance of visionary policymaking and a commitment to sustainable nation branding strategies.

### 2.4 Generating soft power

**Figure 1:**
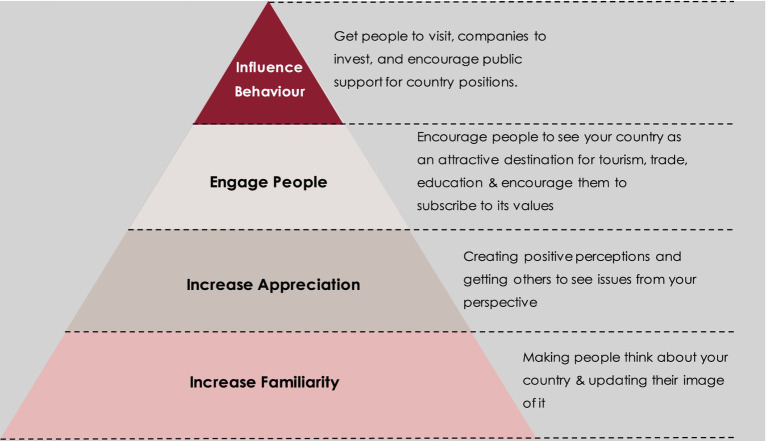
Behavioral influence pyramid. Source: adapted from Leonard and McLaren (2002).

Since soft power derives from attraction and persuasion rather than force, it can be argued that generating non-coercive influence primarily relies on positively framing the mindsets, perceptions and emotions of target audiences in ways that can influence their behaviors. Foreign policy specialist Mark Leonard notes that attracting tourism, increasing inbound investments and trade, and encouraging public support for national positions are examples of national objectives which rely on soft power since audiences cannot, or at least should not, be coerced into visiting, investing or supporting a nation (Leonard and McLaren, 2002). He further reasons that behavioral influence can be achieved through the pillars depicted (Figure 1).

Although not explicitly mentioned in his model, it can be reasoned that ‘encouraging people to subscribe to national values and see the country as an attractive destination for tourism, trade, education etc.’ resembles the concept of soft power as it relies on influencing and persuading foreign audiences to behave in ways that fulfill national objectives, while each of the underlying stages of influence can be achieved through nation branding and/or public diplomacy initiatives. The latter two concepts essentially serve as ‘mechanisms of image-building and building a platform for dialogue and trust… which have been used to describe the process of persuasion’ (Nygård and Gates, 2013, p. 2). Upon examining the various definitions and aims of nation branding and public diplomacy initiatives throughout literature, this paper proposes the following definitions for each:

*Nation branding is the repetitive promotion of simplistic messages, images and information directed at mass audiences to increase familiarity and generate top-of-mind associations regarding a country’s existence, image, identity, and/or competitive advantage*.

*Public diplomacy is the strategic communication and engagement process which conveys complex messages tailored toward target audiences to cultivate understandings, build trust and credibility and foster relationships between governments and foreign publics.*

Given that nation branding aims to increase awareness about a nation, (re)mold its image/identity and promote its competitive advantage(s), it has been reasoned that the former two aims primarily relate to the familiarization pillar of the pyramid as they increased awareness of a country and its image irrespective of others. These initiatives mainly disseminate the name of a country on a global scale, or inform others about the current nature of a country through slogans that foster an awareness of a “friendly” population or a nation that has modernized.

Although the latter aim of promoting competitive advantages can also serve to increase familiarity, it has been reasoned that it primarily relates to increasing appreciation for a nation since it distinguishes a country from others in a way that allows for it to “stand out of the crowd.” As noted by Nye, it is the unique cultural elements of the U.S. that make it seem attractive, exciting and admirable by others. Thus, through promoting a country’s unique culture, business environment, tourism industry, etc., nations can make others not only know about them but appreciate them for matters such as tourism or investment. Therefore, the aims of nation branding can be transposed onto the bottom third of the behavioral influence pyramid.

Overlapping with nation branding in the appreciation pillar are the public diplomacy initiatives which rely on one-way communication channels to explain policy decisions or cultural contexts. It can be argued that through increased understandings, foreign audiences may begin to appreciate a culture that was once considered “closed” or “different” or begin to appreciate policy decisions that they once viewed as rash and unreasonable due to a lack of understanding. Due to the one-way nature of these explanatory initiatives, this part of public diplomacy does not transcend into the engagement pillar.

**Figure 2:**
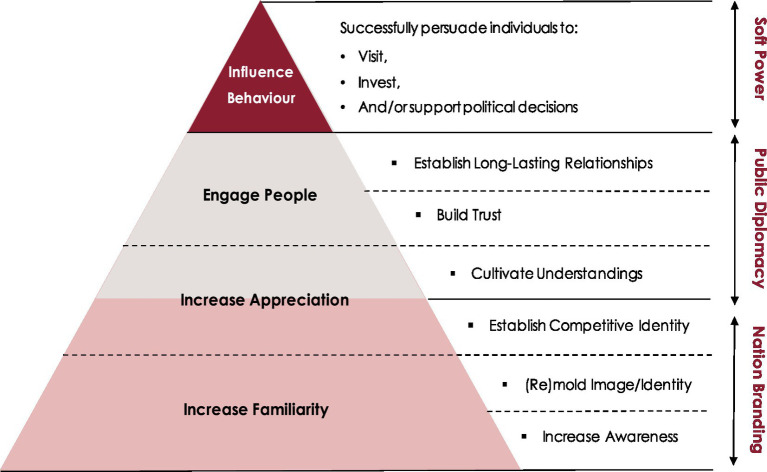
Soft power hierarchy. Source: adapted from Leonard and McLaren (2002).

However, public diplomacy initiatives which consist of two-way exchanges to build trust or cultivate long-lasting relationships have been categorized under the engagement pillar. These exchanges allow for first-hand experiences, the chance to engage in constructive dialogues and are highly subject to individual interpretations. Due to these characteristics, two-way exchanges are viewed as more credible sources than scripted messages conveyed via one-way channels and are more effective in transforming the mind-sets of foreign audiences if managed strategically. Based on these understandings, nation branding and public diplomacy initiatives have been transposed onto the behavioral influence pyramid to develop the Soft Power Hierarchy as follows (Figure 2).

Understanding this phased approach between nation branding, public diplomacy and soft power can help nations establish a holistic strategy toward generating soft power.

## 3 Methods

Qatar has been identified as a prime case study setting to develop a thorough understanding of how sports mega-events can be used to fulfill each of the pillars of the soft power hierarchy, based on the challenging notion of strategic management of a nation. Thus, to develop a ‘holistic’ picture of the soft power framework and correlated objectives outlined above, documentary analysis and semi-structured interviews were conducted to unveil the overarching goals and strategies which must be adopted by Qatar to strategically position a nation brand. This is in line with the suggestions made by qualitative market researchers, investigating branding issues and challenges (Kashif and Udunuwara, 2024).

Since context-driven data was required, the research team opted for a qualitative case study investigation (Edwards and Holland, 2013). In this regard, researchers recommend using triangulation as a useful technique to strengthen the methodology of the study (Kashif and Udunuwara, 2024). An underlying assumption of data triangulation is that ‘data collected in different ways should lead to similar conclusions, and approaching the same issue from different angles can help develop a holistic picture of the phenomenon (Crowe and Sheppard, 2012). It therefore helps guard a researcher against the accusation that their findings are simply an artifact of an unreliable method or source (Patton, 2005). As such, the researchers triangulated the data collected from the document analysis and the semi-structured interviews to ensure consistency of our findings.

### 3.1 Document analysis

The first objective of this study was to understand the pre-determined goals of the nation and the way in which sport event strategies are positioned to fulfill soft power ambitions. Thus, a thorough documentary analysis was conducted as this method is known to ‘*help the researcher uncover meaning, develop understanding and discover insights relevant to the research problem*’ (Merriam, 1988). Depending on the topic at hand, the documents reviewed can range from personal memos and letters to official government documents (Grix, 2010). While it is beneficial to have access to a wide array of documents from different levels and sources, the primary focus should be on the reliability of documents and their relevance to the purpose and design of the study rather than the quantity and breadth of documents (Bowen, 2009).

Due to this latter point, the main focus of this method will be on official government documents relating to the country’s long-term national vision and its respective sport strategies. Thus, national sport strategies and relevant reports and publications were extracted from official government websites. In cases where strategy documents were not made available, however, relevant online articles which disclosed details from interviews with government officials of local sports councils were used in addition to reports provided by personal contacts within local sports organizations. The publication dates and time periods of each document were also accounted for to track any changes in the strategies adopted by Qatar. This helped to determine whether the nation was shifting their focus from internal ambitions to external ambitions.

Despite the relevance of these findings which provide important backgrounds and contexts, policy documents such as these are stagnant in nature as they are published at a specific point in time, are forward-looking and cover a timespan of at least 5-years. Thus, they fail to account for today’s rapidly changing environment unless frequent update reports are released (Bowen, 2009). To address the stagnant nature of document findings and build on the details unveiled throughout this process, semi-structured interviews were conducted with key officials, as advised by qualitative researchers (Patton, 2005).

### 3.2 Semi-structured interviews

Interviews are key to obtaining primary data and can provide researchers with the opportunity to understand the socially constructed realities and perspectives of individuals (Patton, 2005; Kashif and Udunuwara, 2024). Semi-structured interviews consist of a general outline of questions and topics to be covered but is not meant to rigidly guide the discussion (Bryman, 2008). This allows for the interviewee and the respondent to have more liberty in asking and answering questions, but it remains imperative that the interviewer keeps informants on track to obtain relevant answers (Kashif and Udunuwara, 2024).

Semi-structured interviews were therefore employed throughout this study to further build on the findings obtained from the document analysis. A combination of criterion-based sampling which relies on identifying ‘a sample that has the characteristics relevant to the research question’ (Nastasi, 2014) and snowball sampling which ‘typically proceeds after a study begins and occurs when the researcher asks participants to recommend other individuals to study’ were used to identify interview candidates throughout this research (Creswell et al., 2006). The initial approach of relevant interview candidates was facilitated by one of the researchers’ networks. The use of these initial contacts helped establish a sense of trustworthiness amongst the interviewer and interviewees, while also allowing snowball sampling to occur after the interviews were concluded. The research team ensured the maintenance of neutrality in the sampling process by ensuring the criterion-based sampling was met with each interviewee.

It is also important to note that due to familiarity with the mentality of Gulf citizens, the researchers were able to tailor their approach in ways that helped optimize response rates. The mentality of Gulf citizens was taken into consideration when initiating contact with interview candidates. Also, despite being an expat the physical features of the primary researcher closely resemble those of Gulf citizens which enhanced credibility and acceptance amongst interview candidates as it fostered a sense of trust. Rather than being viewed as a “Westerner” looking to gain insider information, the researcher was treated as a local who had genuine interest in bettering the sporting industry of the Gulf. This resulted in more open communication and facilitated their willingness to share information and put the researcher in contact with other interview candidates in the region.

The foundational criteria for interview candidates for the study relied on two main points. The first requirement was that individuals worked within the local sport council, National Olympic Committee, or a local organizing committee of a top-tier sport event within Qatar due to their direct relevance to the objective at hand. The second point was that candidates should be working as a manager or above to maximize the depth of knowledge obtained throughout the interview process. This criteria is inspired by qualitative market researchers, exploring similar issues of brand management (Kashif and Udunuwara, 2024). However, a contingent point was offered in which candidates who were below manager level but had a minimum of 5 years’ work experience in their role could be interviewed due to their relevant expertise and depth of knowledge that may have been obtained over time but may not be represented in a job title such as “officer.” While this criterion was established prior to initiating contact with certain individuals, it also applied to the “snowball sampling” candidates which were recommended by initial contacts (Parker et al., 2019). All interviews occurred face-to-face and relied on notetaking and voice recording based on written consent.

To protect confidentiality and anonymity of interview candidates, participants were informed prior to the interview that their personal and organizational information will remain anonymous and thus verbal and written consent was obtained by the participant at the start of each interview. A coding system was developed to avoid disclosing interviewee names and job titles. The name of each organization and department is provided, however candidates are classified under levels according to their title and/or years of work experience. Officers or individuals with 5 years of work experience will be classified at Level 1; Experts, Senior Officers or those with 7 years of work experience will be classified at Level 2; Managers and those with 10+ years of experience; will be classified at Level 3, and Directors, Board Members, or Executives with 15+ years of experience will be classified at Level 4. Table 1 below is a true depiction of our sample.

**Table 1:** List of interviewees in Qatar.

Qatar interviews organization: name level and department code			
Governing sports bodies	Qatar Olympic Committee	Level 4 Strategic Planning and Project Management	Q1QOC
	Qatar Olympic Committee	Level 4 Marketing	Q2QOC
	Qatar Olympic Committee	Level 4 International Relations/Events Planning & Operation	Q3QOC
	Qatar Olympic Committee	Level 3 Operations	Q4QOC
	Qatar Olympic Committee	Level 3 Operations	Q5QOC
Event specific organizations	2022 Supreme Committee	Level 3 Media Relations	Q6SC
	2022 Supreme Committee	Level 3 Public Relations	Q7SC
	2022 Supreme Committee	Level 2 Media Relations	Q8SC
	2022 Supreme Committee	Level 3 Tournament Operations and Planning	Q9SC
	2022 Supreme Committee	Level 3 Stadium Project Manager	Q10SC
	International Centre for Sport Security	Level 4 Communications	Q11ICSS
	International Centre for Sport Security	Level 4 Planning Coordination and Legal Affairs	Q12ICSS
	Sheikh Khalifa Stadium	Level 1 Project Welfare	Q13SKS
	Sheikh Khalifa Stadium	Level 3 Health and Safety	Q14SKS
	Jusoor Institute	Level 3 Project Management	Q15JI
Other	Qatar University	Level 3	Q16QU

## 4 Results

A thematic analysis approach was used for data analysis. The first step of thematic analysis requires that a researcher familiarizes themselves with the data by reading through it many times so that patterns and themes begin to take shape in their subconscious minds (Spencer et al., 2003). After the “passive” reading process, researchers may begin to generate codes as they “actively” search for meanings and patterns throughout the data sets. Some of these codes may be inductively driven by the data itself, while others may be deductively developed according to *a priori* set of codes that are directly related to the research questions (Crabtree, 1999).

Due to the subjective and interpretive nature of coding, these codes were validated by third parties (Braun and Clarke, 2006). Once codes were generated (and approved by the author team), they were then grouped under broader themes and sub-themes to identify commonalities and differences within and across sources (Gale et al., 2013). Some initial codes may serve as a theme or sub-theme in itself, whereas others may be discarded as they are found to be irrelevant to the main patterns that have been identified from the findings or placed under a “miscellaneous” category (Tuckett, 2005). Each point within these themes were then reviewed and re-adjusted until ‘candidate themes adequately capture the contours of the coded data… [after which] a thematic map can be established from the outcome of this refinement process to display how they fit together, and the overall story they tell about the data’ (Braun and Clarke, 2006).

Themes were inspired by the framework presented above. This deductive and inductive reasoning in which priori themes and new emergent themes from the data were used are the modern ways to construct a pragmatic investigation. A combined approach is appropriate when the project has some specific issues to explore but also aims to leave space to discover other unexpected aspects.

After reading, re-reading, highlighting and annotating each document thoroughly, critical inferences were developed in regard to Qatar’s strategic approach and common themes were noted. This allowed for the researchers to tailor interview guides that specifically addressed the strategies adopted by the nation. Interview recordings were then transcribed using *transcribe.wreally.com* which is an online platform that offers an integrated audio player and text editor within the same page. Following the transcriptions, the authors reviewed the transcriptions to ensure accuracy of the transcriptions. Given that the interview questions were built off document analysis findings, the data obtained from the interview recordings was analyzed in accordance with the pre-determined document analysis framework. This concurrent analysis either validated or corrected for what was noted throughout the examined policy documents and allowed for the researcher to solidify certain inferences.

### 4.1 Document analysis

In October 2008, Qatar launched the Qatar National Vision 2030 (QNV 2030) which outlined the nation’s overarching aim and the fundamental pillars on which the strategy was built. In 2011 and 2019, the country also released 5-year National Development Strategies (NDS reports) to reaffirm or redirect the nation’s focus while also providing updates on the progress that has been made. These reports were significantly more detailed than the 2030 Vision and were useful in understanding the intricacies of Qatar’s overarching vision. In regards to sport-specific documents, the nation has released the 2011–2016 Sport Sector Strategy (SSS) and the 2023–2030 strategy to date. Thus, the following sections will draw on the soft power themes that have emerged from each of the aforementioned documents.

Prior to delving into these details however, it is imperative to highlight that Qatar’s 2030 National Vision begins with the following overarching aim:

*‘The National Vision aims at transforming Qatar into an advanced country by 2030, capable of sustaining its own development and providing for a high standard of living for all of its people for generations to come’ (* General Secretariat for Development Planning, 2008 *, p. 1).*

When reading this statement independently, it is easy for one to infer that the strategy is primarily inward looking as it explicitly notes the intention to serve the local population and future generations. When looking at it through a more analytical lens however, the mere mention of “transforming Qatar into an advanced country” points toward an outward looking and soft power-related ambition. The phrasing implies that Qatar’s current status is lagging behind nations that are considered “advanced” and thus requires a *transformation*.

Upon adopting a thematic analysis to review the details presented within each strategy document, it was found that Qatar is undoubtedly striving to advance its soft power and that sports play a pivotal role in achieving this goal. The nation also demonstrated a rather comprehensive understanding of the key pillars identified within the Soft Power Hierarchy as it was found that the country aims to: (a) increase its international visibility, (b) distinguish itself as a regional cultural hub and a global sporting hub, (c) foster deeper understandings with foreign audiences via dialogues and exchanges, (d) adopt commonly accepted global norms and standards to enhance its credibility on the international front, and (e) increase its cooperation and influence on the international stage while attracting tourism, investments, aid and trade.

#### 4.1.1 Increasing international visibility

When reviewing national documents, it became apparent that Qatar was determined to increase its visibility on the global front. This intention has been expressed manifold throughout the first National Development Strategy (2011 NDS) and the Sports Sector Strategy in particular. In fact, both documents identify sport as a primary way of disseminating Qatar’s name on the international stage. For example, the 2011–2016 National Development Strategy states that: ‘at the international level, sport events and athletes help raise Qatar’s regional and global profile’ (General Secretariat for Development Planning, 2011, p. 202). Echoing these perspectives, the sport strategy documents reason that fostering local talent to participate in world championships such as the World Cup and Olympics, can further disseminate Qatar’s name on the global stage to foster an awareness of its existence. These strategic decisions are directly linked to the awareness objectives of nation branding initiatives that aim to instill a country in the minds of foreign audiences and are in line with the arguments made by previous scholars who have argued that countries use SMEs to “place themselves on the map” and “increase their international visibility.”

#### 4.1.2 Distinguishing Qatar as a regional cultural hub and global sporting hub

As discussed in the literature review, it can be difficult for nations to generate cohesive brands or simplistic messages that cut across various sectors. To address this limitation, they can focus on establishing “sub-brands” that are industry-specific and work toward attracting specified target segments such as tourists or investors which can collectively contribute to the nation’s overall brand. Qatar has clearly adopted this approach by selecting culture and sport as the main sub-sectors in which they aim to distinguish themselves from regional neighbors.

Throughout each document, various explicit references have been made to establishing the nation as a “cultural destination,” a “centre of cultural exchange,” a “cultural hub,” a “sport hub,” a “centre for sport,” and a “first-class sport destination.” Not only are these words used multiple times, but they have been identified as specific targets that have driven strategic decisions as can be seen in the comments below:

*‘Target: Establishing Qatar as a hub of Arab culture through distinctive national identity, strong community cohesion and a vibrant and creative culture’ (2011 NDS).*

*‘[Qatar] is also strategizing to become a global sports hub with an array of first-class facilities and a host of regional and international sporting events’ (2011 Sport Sector Strategy).*

The 2011 NDS further notes that the ‘government will improve branding of the country’s global image using cultural exchange programs and regional arts initiatives to promote Qatar’s culture’, it also emphasizes that a ‘global branding project will be implemented to improve the visibility of Qatar’s cultural sector.’ It is for these reasons that the nation heavily invested in the establishment of a wide array of museums and cultural initiatives to instill Qatar’s heritage within the nation, while also promoting it abroad.

Unlike the Arab culture which may not be universally understood, sports is known for being a universal language that appeals to a wide audience. Establishing Qatar as a “global sports hub” can be classified as a two-way image transfer process known as co-branding ‘whereby image elements from one entity, say for example the Olympic Games which can either be positive, neutral or negative, transfer onto another. In this regard, “sport hub” can serve *as the* brand or as a key facilitator of promoting Qatar *as a* brand. Aside from serving *as the* brand, sport can be a key facilitator of promoting Qatar *as a* brand in itself through disseminating Qatar’s name on the global front as was described earlier.

#### 4.1.3 Foster deeper understandings of the nation via constructive dialogues and exchanges

Further building on these foundations, explicit references are made to the concept of public diplomacy as each document emphasizes the importance of fostering relationships and deeper understandings via constructive dialogues and first-hand exchanges. In fact, one of the key outcomes identified throughout the 2030 Vision is to develop a spirit of tolerance, *constructive dialogue* and openness toward others at the national and international level. Supporting this objective, the 2011 NDS also focuses on the intensification of cultural exchange with Arab people in particular and with other nations in general to create a positive image for Qatar and enhance international relations.

The following passage also demonstrates Qatar’s intentional use of sport to fulfill the public diplomacy aim of relationship-building which has been identified within the soft power hierarchy presented earlier:

*‘International sport is also a powerful tool for international engagement and diplomacy through tourism, aid and trade… and [Qatar] is using sports to forge friendships and improve relations between nations worldwide’ (SSS 2011).*

Based on these comments it is clear that Qatar has adopted the soft power rationale associated with SMEs. It does not shy away from admitting to the underlying intention of using one-way and two-way communication channels that are generated by such initiatives to foster an understanding amongst foreign audiences and perhaps bridge the gap that exists between Qatar and neighboring nations, as well as those from afar.

#### 4.1.4 Enhance trust and credibility by conforming to globally accepted norms and standards

Drawing on the objectives outlined in the 2030 National Vision, the cultural growth section begins with the following quote: ‘the state of Qatar will preserve national heritage and enhance Arab and Islamic values and identity’ (General Secretariat for Development Planning, 2011, p. 204). This point is raised due to the inevitable impact that globalization and popular culture has had and will continue to have on Qatari society (ibid). Furthermore, as the nation attempts to transform and open its doors to expats and foreign tourists, it stresses on the importance of finding a balance between modernization and the preservation of Qatar’s Arab identity.

Despite the intention to retain Qatar’s cultural identity however, the National Development Strategy underlines that ‘social tolerance, benevolence, constructive dialogue and openness toward other cultures’ must also be instilled in the minds of all citizens as ‘the country’s population grows and diversifies, as a new generation ages in a more open society and as private and international interests invest in Qatar’s future’ (General Secretariat for Development Planning, 2011, p. 162).

Further linking this concept to their sport strategy, the nation believes that ‘by developing an array of first-class sports facilities and continuing to host regional and international competitions and events, Qatar will be well positioned to meet QNV 2030’s goal of spirit of tolerance, constructive dialogue and openness through the common sporting principles of teamwork, fairness and aspirational excellence’ (General Secretariat for Development Planning, 2011, p. 196).

Another point clearly exhibiting the point of maintaining credibility through the adoption of commonly accepted norms is the infamous labor rights case that has surfaced since Qatar won the right to host the 2022 World Cup. Again, relating to the concept of soft power, a country’s policies have the ability to generate likeability, respect and admiration amongst foreign audiences which can directly or indirectly lead to influence. However, if a nation adopts controversial policies, this can harm its credibility and lead to resentment or resistance on the international level.

#### 4.1.5 Attracting tourism, investments, and trade and increase cooperation on the international stage

In recent years, Gulf nations have increasingly tried to diversify their economies away from oil revenues. In order to do so, Qatar has prioritized tourism, foreign direct investments (FDI) and trade as ways to diversify its economy. These objectives rely on persuasion rather than coercion as individuals cannot (or at least should not) be forced into visiting, investing or trading with a nation.

Linking these objectives to sport, the 2011 NDS explicitly identifies sport as a powerful diplomacy tool which can attract tourism, investments and trade to lower Qatar’s reliance on oil and gas revenues (General Secretariat for Development Planning, 2011,). Echoing these perspectives, the SSS also states that ‘the provision of quality sports facilities and services, including outdoor recreation facilities, also makes Qatar an attractive place to work, live and visit’ (Qatar Olympic Committee, 2011, p. 14).

The 2030 QNV explicitly states that ‘Qatar will continue to build upon its role in the international community by assuming an increased regional role economically, politically, and culturally, particularly within the framework of the GCC, the Arab League and the Organization of Islamic Conference’ (General Secretariat for Development Planning, 2008,, p. 12). These intentions are directly linked to the concept of soft power as Qatar clearly indicates *how* they intend on becoming influential and relative to *whom*.

### 4.2 Interview analysis findings

As shown in the Table 1, a total of 16 interviews were conducted in Qatar. Five themes have emerged from the interview process, which include: (a) increasing Qatar’s international visibility through sport, (b) fostering deeper understandings amongst foreign audiences, (c) establishing strategic relationships and networks, (d) enhancing Qatar’s credibility as mega- event hosts, and (e) “soft disempowerment.”

#### 4.2.1 Increasing Qatar’s international visibility through sport

As noted in the soft power hierarchy, the first step toward gaining soft power is to generate an awareness about the existence of a nation and its identity which can be done through simplistic messaging and branding techniques that generate top-of-mind awareness across audiences. Throughout the interview process, various candidates expressed that those who resided in countries outside of the Middle East were typically unaware of Qatar’s existence let alone its characteristics. Thus, sport has been identified as a key tool for promotion and publicity, alongside other branding initiatives, due to the competitive advantage it can give the nation. For example, one candidate stated:

*People used to confuse Qatar and not know where it is. Qatar distinguished themselves by using sport because that’s what the other countries did not really go after. Dubai has an economic and tourism distinction and we do not want to compete with them in that sense (Q1QOC).*

The candidate also highlighted that sport is meant to complement other branding efforts that are in place:

*QTA has a strategy in which they try to promote Qatar in every occasion. They even launched the tourism logo in a way that promotes Qatar not only in the region, but internationally. They have their own campaigns and branded taxis in London for example. It’s creating a brand for Qatar. It was a very strategic move on their part to brand the nation (Q1QOC).*

These statements reaffirm Qatar’s explicit intention of branding the nation through sport and using it as a unique selling point. Prior to investing in such branding initiatives and winning the right to host the 2022 World Cup, Qatar lived in the shadows of its regional neighbors as international audiences hardly knew of the country. In recent years however, the country has been at the forefront of international discussions and if it is not known for its national airlines, Al Jazeera/Bein broadcasting network, or investments in football clubs, it is most definitely known as the host of the 2022 FIFA World Cup. Speaking from personal experience, another candidate added:

*Before I came here, when I tell people I’m going to Qatar they ask where is that? If you mention the word Dubai, everyone knew it. The perception is that Qatar was a desert without anything but now everyone knows where Doha is… or almost everyone (Q2QOC).*

Further building on this, and relating to the point of establishing a competitive identity to increase appreciation toward a nation, one interviewee provided the following perspective:

*The main motive earlier, behind Qatar’s bid in the 2000s for the Asian Games was to put Qatar on the map because we all know before then, Qatar only had oil related revenues. They decided to put Qatar on the map through sports events because basically the country could not offer anything more at the time (Q3QOC).*

Worth mentioning here is that this particular statement was made as the interview candidate introduced his position and discussed his experience during the Asian Games. No explicit question was asked regarding Qatar’s motives behind sporting bids, nor its intent to promote the nation’s image or raise its profile. Without probing, another specialist provided the following perspective:

*From a sporting perspective, there are a number of reasons why countries bid and host mega-events. Some bid for major sports events with the intention of using it to leverage the bid to showcase the city or country as a whole: bidding for biddings sake. From the perspective of Qatar as well, obviously bidding and hosting mega-events is a perfect opportunity to bring media to the country to communicate what the country is about (Q16JI).*

These responses point toward a consistent ideology disclosed within the strategy document in which the nation strategically uses sporting events to foster an awareness of its existence and establish a competitive advantage. While one candidate attributes an increased awareness of Qatar to the 2022 World Cup, another one credits the Asian Games (a second-order event) for being the turning point.

#### 4.2.2 Fostering understanding

Throughout the interview process, various candidates expressed the way in which Qatar is commonly misunderstood or misrepresented on the international front particularly since it is associated with negative and outdated stereotypes.

Respondents highlighted that the mere staging of sport tournaments, particularly the 2022 World Cup, is a sign of progress and openness in itself:

*The 2022 World Cup will be the first time the tournament has ever been hosted in an Arab or Muslim country. It is an opportunity for the Arab World to unite and showcase its true, peaceful nature to the rest of the world. It is an opportunity for the region to be in the headlines for reasons other than conflict. It is an opportunity for people to travel to the region and go beyond the stereotypes (Q7SC).*

Similar to BRICS nations that have used first-order events as a way to signal their arrival onto the world stage, Qatar views sport events as a way of signaling its transformation into a more progressive state. Furthermore, through stating that Qatar wants to “be in the headlines for reasons other than conflict,” “go beyond stereotypes,” and “dispel misconceptions and build understandings” as it “can still be looked upon as a closed culture,” it is evident that the nation is struggling from stereotypes that are not in-line with current contexts or intended narratives.

Reaffirming this notion, respondents have also articulated the significance of first-hand exchanges as a way to portray a more accurate picture of the nation especially since the media has painted a distorted picture:

*Misconceptions are created when people just follow news. They’re just following Western news which might not depict an accurate picture of what is truly happening (Q12ICSS).*

*There’s a perception that you’ll be living under same system that KSA are living under for example or the other extreme, the Emirates; but that’s not the case (Q9SC).*

In regards to commonly accepted morals and values, other respondents also reaffirmed that Qatar will continue to preserve its identity, accurately portray it through sport events and also demonstrate that despite the differences, the nation is open, accepting and tolerant toward others as shown in the comments below:

*We are trying to retain and preserve our identity, yet we are leaving room for other cultures to come and showcase what they have. At the end of the day Qatar has its own identity, it is a Muslim/Arab country and there are a few things we cannot get rid of (Q10SC).*

Key stakeholders have absorbed the logic that first-hand exchanges serve as a more credible source of information than the media; however, news travels faster to foreign audiences than audiences do to foreign nations. Thus, Qatar has pinpointed sport as a way of attracting foreign audiences to their nation so that they can engage in first-hand experiences which depict a more accurate picture of the nation and replace the negative or outdated stereotypes that are portrayed via secondary sources.

#### 4.2.3 Establish strategic relationships and networks

Another key point linking SMEs to the concept of public diplomacy is the way in which they can result in the establishment of relationships and networks. Without probing for such answers, interview candidates within Qatar disclosed that sport events were in fact being used to facilitate relationships and networks with pre-determined markets, organizations and businesses. This particular point has actually been a key driver of Qatar’s event selection criteria as one member from the International Olympic Committee (IOC) stated:

*Our strategy was based on a matrix. We wanted to identify federations and events, we selected federations that had the most power within the IOC… we listed their influence within the IOC and listed what major events they have to offer for us to host. We also assessed events based on TV exposure- participation by countries, which countries are most developed? (Q2QOC).*

Instead of selecting events that fulfill inward looking ambitions such as promoting active lifestyles or facilitating the development of advanced infrastructure, the QOC selects events based on their significance within the IOC, their extended reach to a global audience and the participation of developed countries within the tournaments themselves. Each of these criterions can be classified as outward-looking ambitions in which Qatar is striving to engage with external parties. A project manager at Josoor explained that:

*Although workshops are designed for mid-level and above, we gather speakers from around the world and we try to maximize their stay here by tailoring fringe events around the workshops to broaden their impact but also show them more of Qatar (Q16JI).*

Whether it is through bringing international experts to train locals, or through sending locals abroad, Qatar has managed to extend its reach and network beyond its regional premises. It can be argued that these international exchanges were made possible by FIFA’s affiliated network which has allowed for Qatar to engage directly with other nations that have hosted a World Cup in the past.

Aside from Josoor Institute, the International Centre for Sport Security (ICSS) was also established with the primary purpose of transferring knowledge by bringing in international expertise. However, through leveraging its affiliated media network, the presence of this entity has helped Qatar tap into key target markets as explained in the comments below:

*We had mixture of sports journalists and international media from different markets and made sure of showcasing anything the journalists wanted to see in particular such as our new modern convention center (Q12ICSS).*

Thus, the ICSS has allowed for Qatar to leverage its existing network of foreign media representatives to positively showcase the nation in external markets. This can enhance the effectiveness of Qatar’s strategy for two reasons. First, foreign press is likely to have a more “trustworthy” or credible relationship with entities that they have worked with in the past; and second, foreign audiences are more like to accept information from their local media sources rather than Qatar itself as they are deemed as more credible. Another key point that has emerged from these comments are the key target markets that Qatar wishes to engage with to bridge potential gaps that exist due to cultural differences, misunderstandings or a lack of awareness about the nation.

#### 4.2.4 Establishing credibility as mega-event hosts

In addition to increasing familiarity, generating deeper understandings, and fostering relationships through sport mega-events, interview candidates in Qatar also highlighted the importance of enhancing their credibility as mega-event hosts. As noted in an earlier section, countries that host first-order events typically establish their credibility by hosting second and third-order events first. Qatar has also absorbed this hosting mentality as interviewees explicitly stressed on the role of smaller scale events in securing the right to host the 2022 World Cup.

When discussing the selection criteria above, the interviewee pinpointed that the Arab Games was an exception as it did not follow the criteria outlined in the strategy matrix:

*In 2010, we hosted the Arab games which was a unique story, it followed a different strategy. During 2009 and 2010, Qatar was bidding to host the 2020 Olympics. To do that we wanted to show the IOC that we have the capabilities to host an Olympic-like multisport event (Q2QOC).*

By labeling this as a unique story and one that followed a different strategy, it can be inferred that the Arab Games is not viewed as a tournament that is “significant” within the IOC; and due to its regional scope, it does not have the wider global reach that Qatar wants nor does it involve the developed countries that Qatar would like to engage with. Relating to this, another candidate stated:

*The Arab Games showed the region and the IOC what we could do. I think it really raised our profile a lot and at the same time we were bidding for other events with the federations. As those federations came and saw the work and facilities and hospitality that we have, they knew about us (Q4QOC).*

In addition to the Arab Games, other interviewees stressed on the importance of using the Handball Tournament, Asian Games and other events as a stepping stone to establish Qatar’s reputation as credible mega-event hosts and also to build their hosting expertise:

*Before the Asian Games, Qatar hosted smaller events to build the experience, the profile, etc. There have been more than 40–50 events a year and it maintains also what we started with the Asian Games. The Asian Games to me is what turned Qatar around. It proved Qatar can organise sports events and go to any length needed to have such tournaments (Q3QOC).*

Interviewees from each organization have expressed the way in which Qatar’s labor force has benefitted from hosting a wide array of mega-events over the years. Initial tournaments relied on a higher number of foreign expertise, but with time, the transfer of knowledge to Qataris has led to a rise in the number of locals working in Qatar’s sporting industry. Thus, the country has slowly managed to enhance their capabilities internally and readjust perceptions externally through the hosting of second and third-order events.

### 4.3 Theoretical model as an outcome

Understanding these characteristics helped unveil the way in which soft power, public diplomacy and nation branding fit into the Soft Power Hierarchy. This theoretical model served as a guide to analyze how Qatar utilized sports mega-events to enhance its soft power. Throughout the strategy documents and interview process in Qatar, various comments were made in regard to using sport to emerge from the shadows of its local/regional neighbors (namely Dubai and Saudi Arabia) and to correct for negative/outdated stereotypes. Qatar’s strategy was explicitly linked to the concepts of nation branding, public diplomacy and soft power which exhibited that the country had a clear understanding of the relationship between sport events and each of the soft power components. As summarized in Table 2, sport events were being used to place the nation on the map, signal its transformation, establish its competitive identity as a sporting hub, showcase its culture, enhance its credibility, and foster long-lasting relationships to extend its global influence and increase inbound tourism and trade, all of which have been highlighted as key components of the Soft Power Hierarchy. Thus, Qatar serves as a prime example of an emerging state that is using sport to extend its international influence through adopting a holistic approach.

**Table 2:** Qatar document analysis and interview findings in relation to soft power hierarchy.

	Qatar		
	Soft power hierarchy pillars	Doc analysis	Interview analysis
Soft power	Attract Tourism	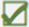 Attract international tourists particularly via sport events	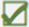 Primarily target audiences from developed nations
	Attract Investment/Trade/Business	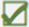 Diversify economic revenues and attract FDI	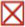 N/A
	Obtain Political Support	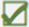 Increase regional and international cooperation and influence	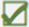 Events associated with influential federations in IOC, events in which developed & loudest countries participate
Public diplomacy	Establish Long-Lasting Relationships	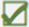 Build strategic partnerships and business networks	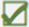 FIFA affiliated network, ICSS affiliated network, Jusoor network, etc.
	Build Trust/Credibility	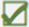 Instill values of tolerance, benevolence & openness, & reform labor laws to conform to international standards	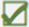 Showcase hosting capabilities by hosting smaller scale events, open to other cultures, ammended labor laws
	Cultivate Understandings	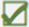 Engage in exchanges to foster deeper understandings	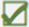 Exchange programs, showcase Qatari culture, tackle negative criticism
Nation branding	Establish Competitive Identity	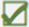 Sporting hub and Cultural hub	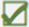 Sporting hub
	(Re)mold Image/Identity	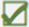 Modernized and developed nation	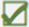 No longer a desert, transforming
	Increase Awareness	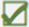 “Place Qatar on the map”	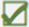 “Place Qatar on the map,” brand the nation

Source: The table has been developed by the authors.

## 5 Discussion and conclusion

Based on the document analysis, it has become evident that Qatar clearly intends on operating across all the levels identified in the soft power hierarchy. Although the documents do not present these initiatives in the sequential order of the soft power hierarchy, they each acknowledge the need to establish a brand and fostering an awareness of the nation via one-way image-projection channels (nation branding). They also place a special focus on hosting cultural exchanges and sporting events to engage in constructive dialogues and foster deeper understandings between countries, both of which fall under the public diplomacy umbrella. The country has also outlined its intent to adopt commonly accepted norms and values and conform to international standards to enhance its credibility amongst foreign audiences. It is only upon doing so that the country can position itself as an influential economic, political, and cultural leader within the region and begin to extend its influence to an international audience.

Each of these objectives have been linked to sport as well as the country explicitly states its overt intention to use sport as a nation branding and public diplomacy tool to enhance its soft power. Sports events and athletes have been identified as a way to “raise the nation’s regional and global profile,” international sport events have been labeled as a “powerful tool for international engagement and diplomacy” and credited for “forging friendships and improving relations between nations”; and the establishment of sporting facilities and hosting of mega-events has been linked to making Qatar an “attractive place to work, live and visit.”

Despite the internal benefits that sport may bring to the nation, it has also become evident through interview responses that through hosting mega-events, the nation ultimately aims to fulfill external objectives relating to the concepts of nation branding, public diplomacy and soft power. Respondents expressed the way in which Qatar has managed to successfully put itself on the map through hosting mega-events, and that branding the nation was a primary driver behind their sport event strategy.

At the public diplomacy level, the event selection criteria were strategically tailored to select events that are popular in the developed countries with which Qatar seeks to further engage an develop relationships, and their affiliated marketing mechanisms were also directed at establishing a dialogue with these audiences in hopes of creating and readjusting their perceptions through first-hand visits. Furthermore, the nation intentionally selected events based on their significance and potential influence in the IOC. The constant need to be affiliated with advanced nations and influential powers at the political and sporting level points toward Qatar’s soft power ambition of rising above its “small-state status” to be seen as a global player and one of the “big guys” regardless of its size.

Upon successfully doing so, the nation realized the way in which the negative press and cultural divide between Western and non-Western nations may impact its ability to attract spectators and hinder its reputation. Therefore, various measures have been implemented to bridge these gaps and create an understanding amongst foreigners that Qatar is in fact open to tolerating different cultures while maintaining its own traditions, and that it is a friendly, warm and hospitable nation contrary to the alleged conservative environment it hosts and the alleged negative terrorism and labor rights reports that have surfaced in recent years. The Gulf Crisis, between Qatar and the Kingdom of Saudi Arabia between 2017 and 2018, has also posed an additional challenge for the nation as it has severed ties between regional neighbors and impacted Qatar’s reputation on the global front. Nonetheless, the nation remained relatively optimistic that the 2022 World Cup will not be impacted by the Gulf Crisis and that building bridges with those from developed countries and Western nations remains their primary objective as they are the main target audiences.

There are several interesting outcomes based on the findings of data collected for the purpose of this study. The discussion of these findings is classified into three broader elements. First, soft power can be exhibited through attracting tourism toward nations. This is in line with the findings of previous studies where attraction of tourists has helped nations in establishing and to strengthen their image (Avraham, 2020). However, a more focus on attracting sports tourism is a unique aspect of this study, underexplored by previous researchers. Another manner which evolved from the data is via investment in business and trade. There are several studies where investment and trade options are outlined to develop nations. However, from the perspective of our study, these opportunities can help in establishing soft power for nations. In this way, our study contributes to previous findings which were focused more on organizations rather than nations (Sertyesilisik, 2021; Grewatsch et al., 2023). Extending on this point, it also paints an image of strategic intent which is required among policymakers to chalk-out strategies which establish strong national image. Another element of soft power is bilateral ties/relationships with other nations. There is a strong need felt to collaborate with other country states. However, slightly diverging from the extant literature, we position political support to collaborate in a more holistic yet sustainable manner which can outline a positive way forward.

We have reflected on the potential of mega-sport events to enhance a nation’s soft power based on increased global visibility, enhanced international relations, and competitive business advantages (Anholt, 2007; Leonard and McLaren, 2002). Venturing deeper, we more profoundly surface the internal and regional impacts of hosting sports, including creating community pride, promoting cultural exchange, and enhancing the nation branding. Events like the 2022 FIFA World Cup in Qatar illustrate how nation branding and public diplomacy intertwine to present a modern and progressive identity, dispelling outdated stereotypes and reinforcing regional solidarity (Nye, 2004; Szondi, 2008). In the Arab world, such investments not only serve economic goals but also project values of cultural richness and unity, deploying soft power to strengthen both internal cohesion and global influence (AlBuloushi et al., 2024; Grix and Brannagan, 2016; Mattern, 2005). The accomplishments of Qatar as nation increasing in prominence on the global stage underscore the strategic role of mega-events in reframing narratives and advancing sociocultural and political aspirations n a highly competitive global attention arena.

While sport and hosting mega-sport events represent noteworthy tools of soft power for Qatar, they are part of a broader, multifaceted strategy. Qatar also uses cultural diplomacy, education initiatives such as the establishment of global university campuses, media platforms like Al Jazeera, and diplomatic networks to enhance its soft power reach (Nye, 2004; Szondi, 2008). We note these complementary approaches alongside the use of mega-sport events to provide a balanced understanding of how Qatar strategically projects its influence on the global stage. The synthesis of approaches indicates a commitment to cultivating a well-rounded and resilient soft power identity.

Another important domain is public diplomacy which plays a key role in building mutual trust and friendly relationships among nation states (Daßler et al., 2019; Sertyesilisik, 2021). A key feature of this element is strategic thinking, based on the principles of strategic management (i.e., setting long-term goals, thinking holistically, outlining an inspiring vision, and implementing and learning from the implementations). An important part of public policy is openness toward others which is not frequently discussed in extant nation branding literature, particularly in instances where a strategic management agenda inspires implementations. Reinforcement is particularly used by brand marketers to strengthen brands (Avraham, 2020) however, our agenda is more macro in its perspective, thus, steering away from the extant literature. A key to partnerships is a strong reinforcement strategy which supports dialogue at all levels. This includes political leaders and even strategists, engaged in the process of implementing the strategies. This way, it can deepen understanding among key stakeholder groups.

The 2022 FIFA World Cup represents a significant case for examining the impact of nation branding on soft power, particularly in the Arabic-speaking world and the GCC region. Prior to the event, sentiments were mixed, with criticism centering on the suitability of Qatar as a host due to its size and cultural differences (Nye, 2004). However, during the event, extensive cultural diplomacy efforts and Qatar’s emphasis on its Arab identity helped foster regional pride and solidarity, shifting perceptions positively among neighboring nations (Grix and Brannagan, 2016; Szondi, 2008). Post-event analyses indicate that Qatar relied on and benefited from the World Cup not only to enhance its global visibility but also to bolster regional unity and reshape its image as a hub for cultural and sporting excellence (Anholt, 2007). Comparing public sentiment across these time periods highlights how strategic soft power initiatives can alter perceptions over time.

Finally, another theme which is emphasized as part of this study is nation branding. Although much has been said about it, nation branding from our perspective is missioned around building a desirable global image of a country, inspired by elements of the national identity, while polishing the existing stereotype and heightening stakeholder awareness. Various sports can help build this new image for Qatar and to steer away from unjustified stereotypes. Such a transformation becomes possible when policymakers understand key components of nation branding, interwoven with strategic thinking, and forging a way forward to develop and implement a sustainable soft power-based agenda. With such insights, our study extends and amplifies extant research on nation branding (Rojas-Méndez and Khoshnevis, 2023).

In conclusion, our study has highlighted Qatar’s strategic use of sport as a central tenet in advancing its soft power, nation branding, and public diplomacy. By leveraging globally renowned sports mega-events to attract international audiences, build multilateral relationships, and intensify intercultural understanding, Qatar has developed a capability to mine the complexities of global perceptions while also handling challenges such as the Gulf Crisis and periodic negative media coverage. The findings emphasize the importance of strategic intent and holistic public policymaking in reshaping national identity and bridging cultural divides. Qatar’s example underscores how integrating strategic management principles with nation branding efforts can transcend traditional approaches, positioning a nation as a competitive and advantageous player on the global stage. Ultimately, our research contributes to the broader understanding of how soft power, grounded in culture, diplomacy, and strategic vision, can transform a nation’s global image and redefine its role in international relations.

## Data Availability

The original contributions presented in the study are included in the article/supplementary material, further inquiries can be directed to the corresponding author.
